# Not as information, but as a trigger: recurrent climate trauma after the 2024 Rio Grande do Sul floods

**DOI:** 10.1016/j.lana.2026.101593

**Published:** 2026-08-03

**Authors:** Samuel Paulo Cibulski

**Affiliations:** Faculdade de Ciências da Saúde do Trairi (FACISA), Universidade Federal do Rio Grande do Norte (UFRN), Santa Cruz, 59200-000, RN, Brazil

My father, who lives in Eldorado do Sul, the city most affected by the 2024 floods in Rio Grande do Sul, has been showing signs of post-traumatic stress disorder. During a discussion with neighbours about protective measures against possible new floods, fearing the effects of a potentially strong El Niño in 2026,^1^ he experienced a spike in blood pressure. Many neighbours already exhibit signs of anxiety and depression that arose after the loss of everything they had: homes, crops, animals, memories.

In May 2024, the worst climate disaster in southern Brazil affected nearly 2·4 million people, displacing more than 580,000.^2^ Two years later, the population exposed to the disaster shows symptoms of moderate-to-severe anxiety and depression at rates 36% and 72% higher than expected, rising to 77% and 129% among the most affected.^3^ Post-traumatic symptoms intensified over time, evolving from hyperarousal to re-experiencing and avoidance.^4^

However, there is a characteristic of trauma related to climate disasters that research and public policies neglect: this threat does not end. Worse still, as climate change advances, it becomes increasingly frequent. In flood-exposed cities, weather alerts can act as trauma triggers, generating anticipatory distress. Communities like my parents’ already face heavy rains and the imminence of El Niño with collective hypervigilance. In neighbourhood gatherings, the topic is always the same: the disaster, the losses, what to do when the waters return.

Most trauma treatments assume that the threat has passed. For my father and his neighbours, it has not passed and could return at any moment. Despite calls made soon after the 2024 disaster to integrate mental health into disaster response in southern Brazil,^5^ implementation is still lagging behind. Unmet need for psychological support predicted the severity of symptoms more strongly than flood exposure itself.^2^ Care remains concentrated in the acute phase and in large urban centres, a common pattern in the Global South, where resources are scarce precisely in peripheral and repeatedly affected communities. Smaller municipalities rely on primary care teams without training in disaster mental health.

To break this cycle, permanent psychosocial care programmes must be part of climate change adaptation plans. These programmes must go beyond the immediate response to floods, training and supporting primary care teams to recognise and manage prolonged and anticipatory trauma, including protocols for periods preceding predicted extreme weather events. Without these measures, each new weather forecast will be received not as information, but as a trigger.

## Declaration of generative AI and AI-assisted technologies in the manuscript preparation process

During the preparation of this work, the author used Claude (Anthropic) to assist with grammar and spelling of the manuscript. The author reviewed and edited the output as needed and take full responsibility for the content of the published article.

## Declaration of interests

I declare no competing interests. I write as a native of Rio Grande do Sul whose family and home community were directly affected by the 2024 floods.
